# Tomato leaf dataset: A dataset for multiclass disease detection and classification

**DOI:** 10.1016/j.dib.2025.111520

**Published:** 2025-03-27

**Authors:** Ahmed Imtiaz, Fahad Bin Islam Swapnil, Syed Rayhan Masud, Debajyoti Karmaker

**Affiliations:** a Department of Computer Science, American International University-Bangladesh, Dhaka 1229, Bangladesh; b Faculty of Agriculture, Khulna agricultural University-Bangladesh, Khulna 9100, Bangladesh

**Keywords:** Tomato leaf classification, Disease prediction, Deep Learning, Image annotation, Machine learning in agriculture, Image processing in agriculture

## Abstract

Agriculture is a cornerstone of Bangladesh's economy, with tomatoes being one of the most widely cultivated vegetables, producing approximately 368,000 tons annually. However, tomato plants are vulnerable to various diseases and pest infestations that can significantly reduce crop yield, posing a threat to farmers’ livelihoods. Early detection of these diseases, often visible through symptoms on the leaves, is critical for effective management. In this work, we present a dataset of 731 high-resolution images of tomato leaves affected by six common diseases, along with healthy samples, aimed at facilitating automated disease diagnosis using computer vision. The dataset is categorized into disease types such as Early Blight, Black Spot, Late Blight, Leaf Mold, Bacterial Spot, and Target Spot. This structured dataset offers a valuable resource for researchers developing machine learning models for disease classification and early detection. By making the dataset publicly available, we aim to accelerate research in precision agriculture and empower the development of AI-driven tools that can enhance tomato disease management, ultimately improving crop yields and supporting sustainable farming practices.

Specifications Table

**Table:** 

Subject	Agricultural Sciences, Computer Science
Specific subject area	Computer Vision, Image processing, Deep learning, Agriculture.
Type of data	Raw: jpg, Annotation: TXT
Data collection	The data for tomato leaves were collected from tomato gardens in Dina- jpur, Thakurgaon, and Kushtia. An experienced gardener from Kushtia and workers from other tomato gardens provided important insights about tomato leaves. Over seven days, we captured around 731 pictures of all categories and classified them according to their types.
Data source location	Tomato is one of the most commonly cultivated vegetables in Bangladesh. We collected our data from these three locations: 1. Dinajpur 2. Thakurgaon 3. Kushtia
Data accessibility	The dataset is published in Mendeley Data. • Data identification number(doi): 10.17632/bpfd9cns5g.2 • Direct URL to data: https://data.mendeley.com/datasets/bpfd9cns5g/2

## 1 Value of the Data

The dataset is highly valuable for agricultural research and machine learning applications, especially in tomato cultivation, providing valuable insights and potential impacts.

• The Tomato Leaf Dataset [2] provides a comprehensive collection of tomato leaf, images, categorized by disease type, aiding in precise research on tomato leaf diseases, aiding in understanding and predicting disease progression from visual symptoms.
• The “Tomato Leaf Dataset” clear categorization of disease classes makes it an ideal resource for developing deep-learning-based detection algorithms, enabling researchers and developers to design and train models for automatically detecting and classifying tomato leaves.
• Treatment practices influenced by disease progression can be improved by understanding the correlation between visual symptoms and disease, thereby enhancing crop yields and guiding researchers in determining optimal intervention times for optimal plant health.
• Future studies will utilize this dataset for comparative research on tomato leaf diseases across various regions, climates, and farming practices. This will enable researchers to validate their approaches and explore regional variations in disease prevalence and impact.

## 2 Background

Tomato cultivation is a cornerstone of Bangladesh's agricultural sector, both in terms of domestic consumption and as an export commodity [1]. With an annual production of approximately 368,000 tons, tomatoes are a vital crop that significantly contributes to the livelihoods of farmers across the country. In 2022, Bangladesh exported $64.7k worth of tomatoes, with major destinations including Malaysia, Singapore, the United Arab Emirates, Poland, and the Maldives. Given the increasing demand for tomatoes both locally and internationally, ensuring the health and productivity of tomato crops is of paramount importance for the country's agricultural economy. Despite their importance, tomato plants are highly susceptible to a variety of diseases, many of which can severely impact yields and cause significant economic losses. Diseases such as bacterial spots, early blight, and fungal infections are common in Bangladesh's tomato fields, often exacerbated by the country's tropical climate. The early detection and management of these diseases are essential to mitigate their effects, and the most reliable indicators of infection are typically visible on the leaves. Observing leaf symptoms allows for the diagnosis of diseases at an early stage, providing an opportunity to intervene before the damage spreads to the entire plant or field. Traditional disease detection methods involve manual inspection by agricultural experts or farmers, which can be time-consuming, labor-intensive, and prone to human error. These conventional approaches are not always feasible, especially in large-scale farming, where early-stage symptoms may go unnoticed until the disease has already spread. The need for faster, more accurate methods of disease detection has led to increased interest in leveraging modern technologies, particularly in the fields of computer vision and machine learning. Recent advancements in artificial intelligence (AI) [3] and deep learning have revolutionized precision agriculture, allowing for the automation of tasks that previously required manual labor. Computer vision models, trained on large datasets of plant images, are increasingly being used to detect and classify plant diseases based on visual symptoms. By automating the process of disease detection, these models can significantly reduce the time required to diagnose problems, leading to quicker responses and more effective management strategies [3]. Tomato leaf-related datasets have played a pivotal role in advancing the field of plant disease detection, enabling the development of accurate and efficient diagnostic systems. These datasets vary in scope, composition, and application, each contributing uniquely to the broader goal of improving crop yields and reducing losses due to diseases. This section discusses the contributions and novelty of various tomato leaf datasets, highlighting their significance in the context of deep learning and machine learning applications. One of the most notable contributions of tomato leaf datasets is their diversity in terms of disease categories, image quality, and geographical representation. For instance, the PlantVillage dataset, widely used in several studies, includes images of healthy and diseased tomato leaves, covering diseases such as bacterial spot, early blight, septoria leaf spot, and yellow leaf curl [4,6,10]. Similarly, the Taiwan dataset focuses on specific diseases like bacterial spot, early blight, and leaf mold, providing a localized perspective on tomato leaf diseases [9]. These datasets are often combined with custom-collected images to enhance their robustness and applicability to real-world scenarios [7,8]. The inclusion of high-resolution images, as seen in studies utilizing the EfficientNetV2B2 model, ensures that fine details of leaf textures and disease symptoms are captured, enabling more accurate classifications [5]. Additionally, datasets like the one used in the Siamese network-based framework are designed to handle imbalanced and small datasets, addressing a common challenge in agricultural image classification tasks [11,12]. In this study, a dataset comprising 731 high-resolution images of infected tomato leaves has been compiled to support the development of computer vision models for tomato disease detection. These images capture a variety of leaf diseases, each with distinct symptoms, providing a robust dataset for training and testing AI algorithms. The use of such a dataset is crucial for developing models capable of accurately identifying diseases, even in the early stages of infection. The integration of machine learning models into tomato farming practices could enhance decision making processes by providing farmers with real-time disease diagnostics, ultimately improving yield and reducing the economic losses associated with crop diseases. This research aims to bridge the gap between traditional farming practices and modern technological solutions, addressing the specific challenges faced by farmers in Bangladesh. By utilizing digital technologies, such as AI and deep learning, the study seeks to streamline disease detection and provide a cost-effective, scalable solution for the agricultural community. The development and adoption of these technologies are expected to play a critical role in the future of sustainable farming, not only in Bangladesh but also in other agriculture-dependent economies.

## 3 Data Description

**Fig. 1:**
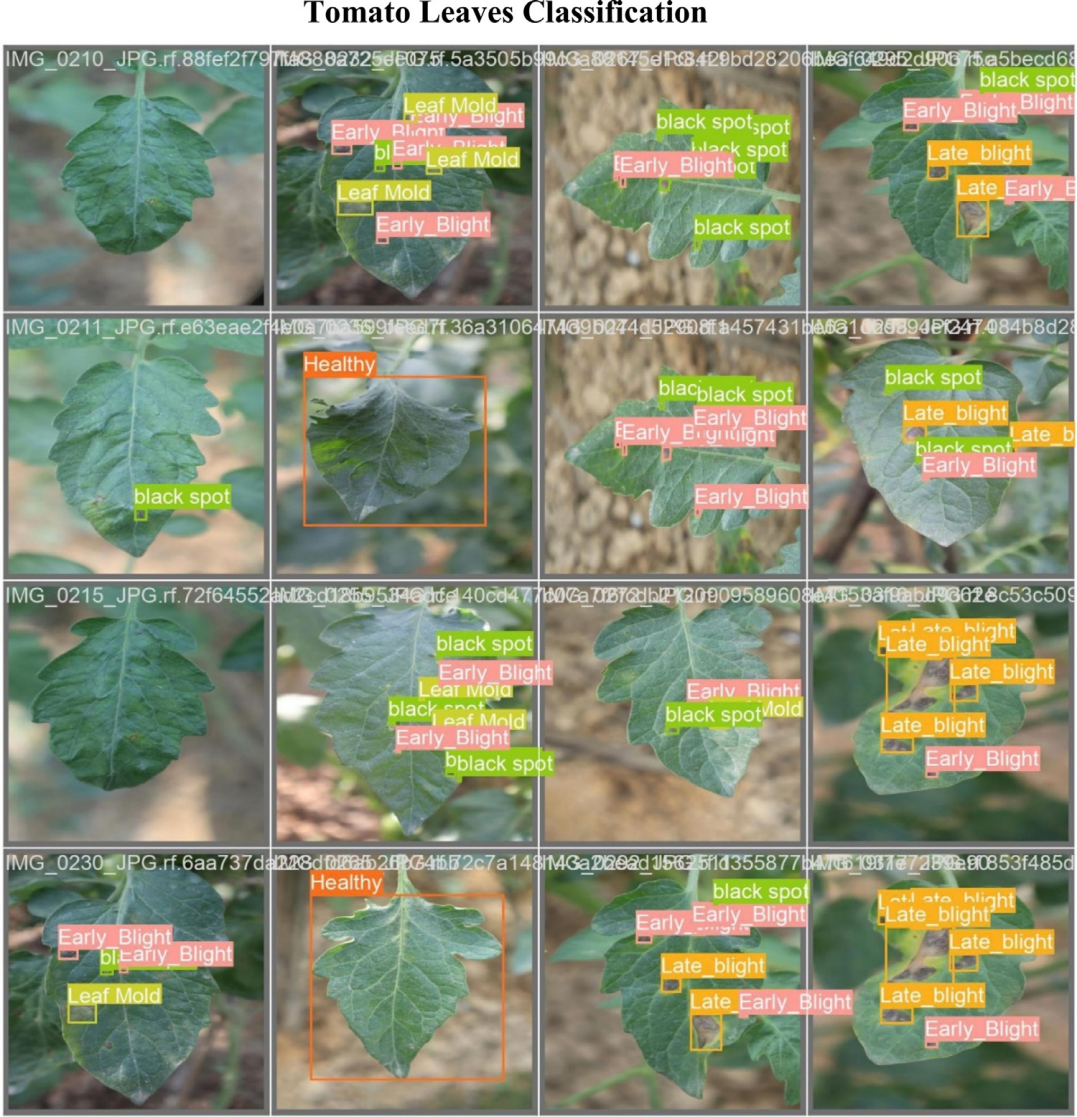
Sample images of the 7 categories of the Tomato Leaf Multiclass dataset.

The “Tomato Leaf Dataset” is astutely organized into seven types, each type representing a particular tomato leaf to streamline get to and examination. The first directory, “Tomato Leaf Multiclass (Raw Data), These raw pictures, sourced from different tomato areas, capture the side effects of six essential diseases. Early Blight, due to Alternaria solani, shows up as dim brown spots with concentric rings on more arranged takes off, causing veritable defoliation and abdicate occurrence. Black Spot, caused by diverse Alternaria species, presents as small gloomy spots that will mix into greater patches, affecting photosynthesis and plant vigor. Late Blight, from Phytophthora infestans, spreads quickly with water-soaked injuries and white form, competent of obliterating crops in the event that unmanaged. Leaf Mold, caused by Passalora fulva, is recognized by pale spots on the upper leaf surface and shape underneath, which can result in less than ideal leaf drop. Bacterial Spot,from Xanthomonas campestris pv. vesicatoria, appears up as little, water-soaked wounds with yellow halos, essentially influencing the foliage and characteristic thing quality. Target Spot, caused by Corynespora cassiicola, highlights melancholy concentric spots and impacts leaf success, driving to decreased photosynthesis and leave. Finally, Healthy leaves show no signs of disease, providing a baseline for comparison to ensure accurate disease identification. Each category, containing a collection of raw pictures, is housed in its organizer inside the dataset, publicizing an organized foundation for examination. Table 1 shows the numbers of annotation of every class, whereas Fig. 1 gives visual cases of each disease and healthy leaves.

**Table 1:** Description of annotation data by tomato leaf.

Class No.	Class	No. of Annotations
1	Early Blight	325
2	Black Spot	254
3	Late Blight	202
4	Leaf Mold	194
5	Bacterial Spot	233
6	Target Spot	213
7	Healthy	200
	Total	1621

**Fig. 2:**
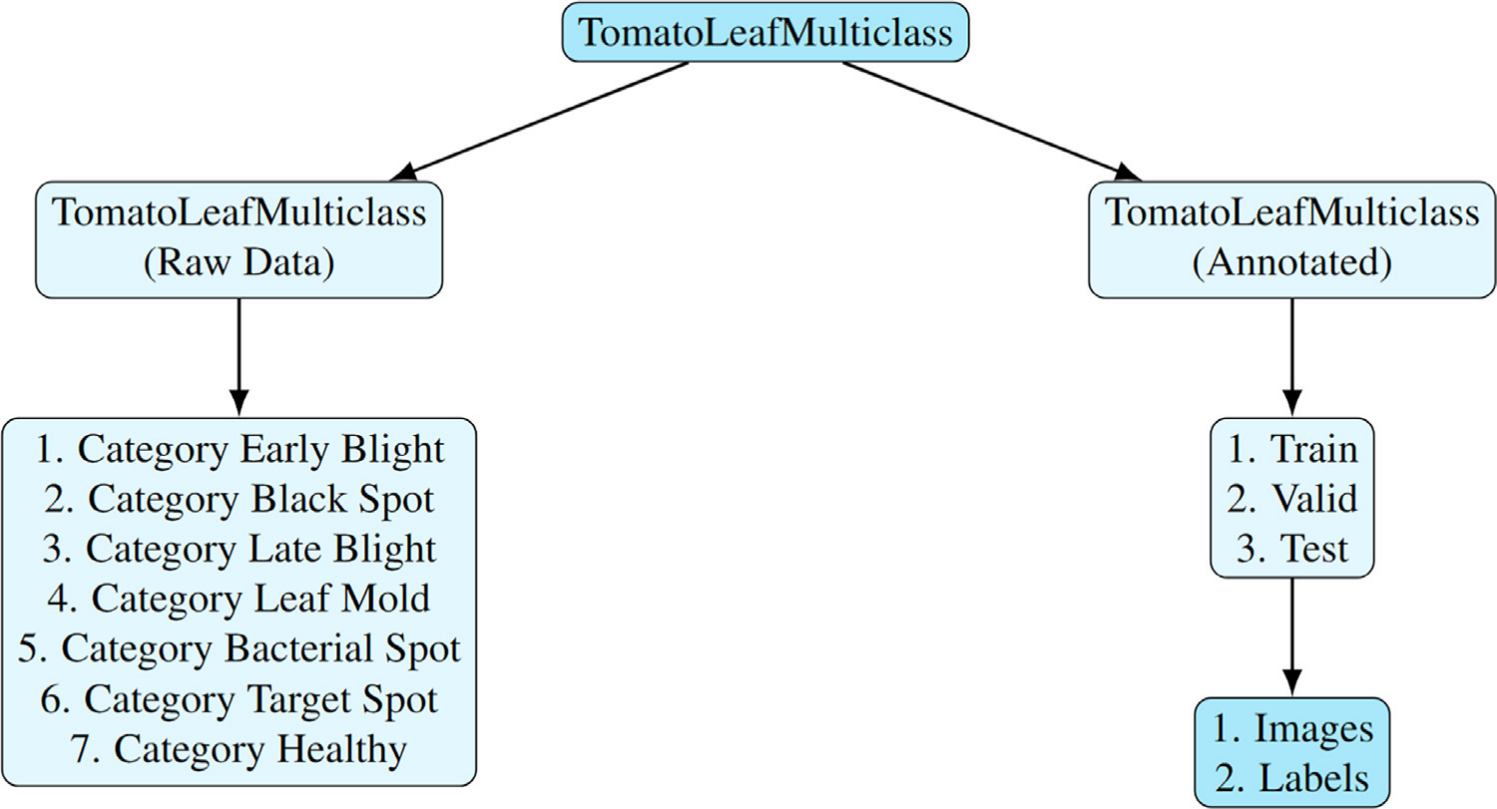
Hierarchy of the Tomato Leaf Multiclass dataset directories.

The identical photos from the raw data are included in the second directory, “TomatoLeafMulticlass (Annotated),” along with annotations. Roboflow, an efficient tool that improves the dataset's accuracy and usability, was used to make these annotations. For researchers who must do point by point analyses and develop models based on the various circumstances of tomato leaves, this annotated dataset is essential. Two subfolders are present in the “TomatoLeafMulticlass (Annotated)” directories: train, test, and valid. These subfolders provide a systematic method for training, validating, and testing models by containing photos and their corresponding annotation names. The “TomatoLeafMulticlass” dataset is guaranteed to be a valuable and adaptable resource for scientists and researchers working to advance the fields of tomato leaf disease prediction and classification by this all-inclusive organization. Fig. 2. shows the overall organization of the dataset directories.

## 4 Experimental Design, Materials and Methods

**Fig. 3:**
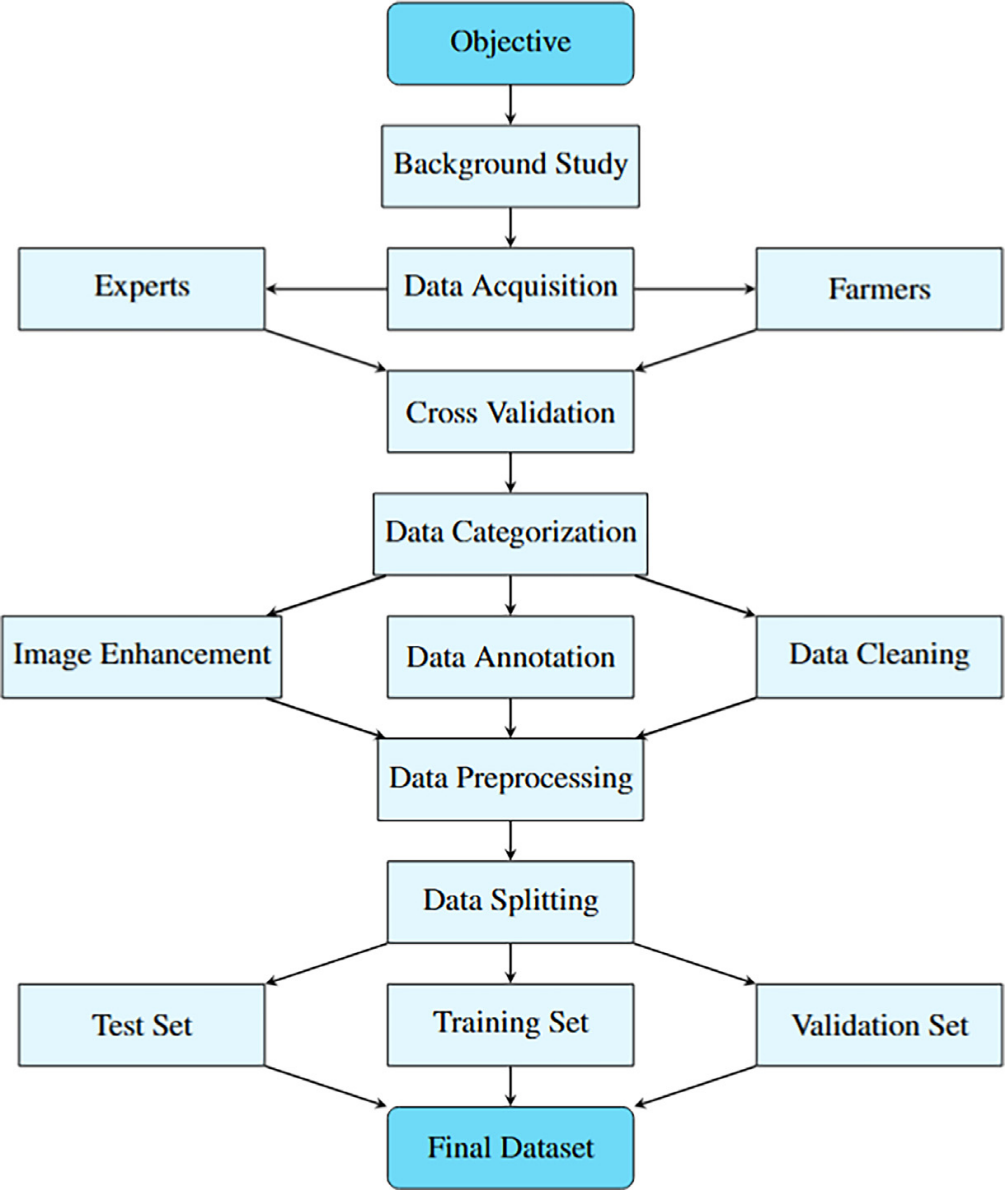
Workflow of the Tomato Leaf multiclass dataset creation.

The general experimental design and dataset creation methodology are covered in this section. Fig. 3 illustrates this process.

### 4.1 Objective

The dataset aims to develop machine learning models for accurately recognizing and classifying diseases in tomato leaf images, promoting early detection and management of plant health issues.

### 4.2 Location

**Fig. 4:**
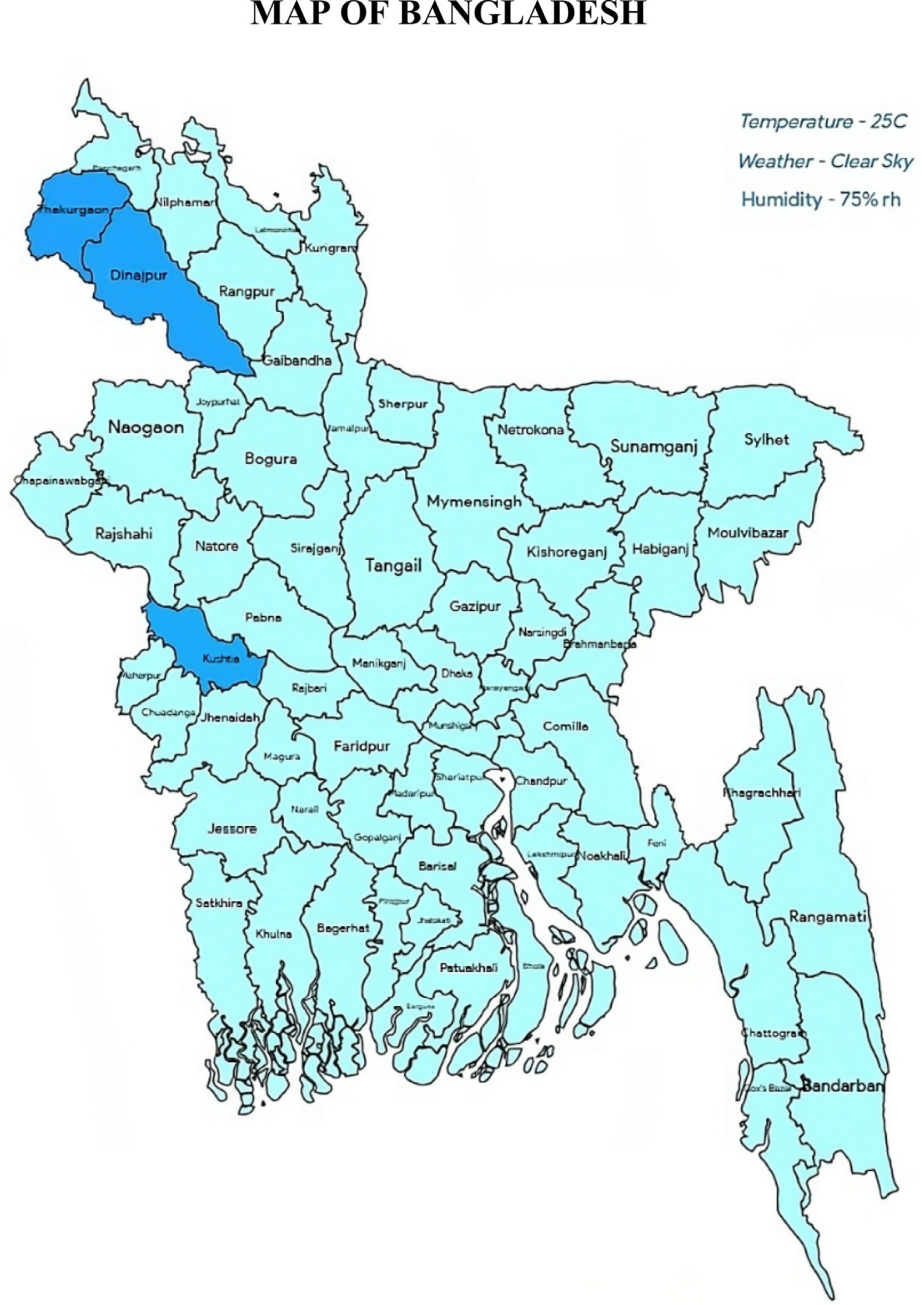
Major source of data acquisition zones.

The major source of data was the tomato gardens in Dinajpur and Thakurgaon, with additional data provided by tomato gardeners in rural Kushita. Fig. 4. shows the major source of data acquisition zones in Bangladesh.

### 4.3 Sampling methodology

Tomato leaves were categorized into seven classes based on disease symptoms and visual quality indicators, including Target Spot, Bacterial Spot, Early Blight, Leaf Mold, Late Blight, Black Spot, and Healthy. These categories were included in a multi-disease analysis for comprehensive dataset coverage.

### 4.4 Data collection process

#### 4.4.1 Camera device

The Canon EOS M50 high-resolution camera was used to efficiently capture images of tomato leaves, ensuring easy access and efficient collection.

#### 4.4.2 Engagement with experts

Initial discussions with field workers and agricultural experts provided foundational knowledge and validated the types and symptoms of tomato leaf diseases.

#### 4.4.3 On-Site collection

We visited various tomato fields to capture images of tomato leaves exhibiting different diseases. The collection spanned several days to ensure a diverse representation of the conditions and stages of disease progression.

### 4.5 Data processing and annotation

#### 4.5.1 Software tools

To ensure accuracy and consistency in labeling the images, the annotation process was conducted using Roboflow, a robust tool designed for object detection, classification, and segmentation tasks. Each image was meticulously annotated according to predefined disease categories, ensuring clear classification for machine learning model development. Roboflow's intuitive interface streamlined the annotation process and enhanced the overall usability of the dataset.

#### 4.5.2 Image scaling

To optimize the dataset for efficient processing in machine learning models, the raw high-resolution images were resized to a standard dimension of 640 × 640 pixels. This uniform scaling ensures that all images maintain consistent quality while reducing computational complexity. The resizing process was critical in ensuring the dataset's compatibility with a variety of machine learning algorithms without compromising image clarity. To resize an image with original dimensions (H, W) (height and width) to new dimensions (H′, W′) while maintaining the aspect ratio, the scaling formula is given by:

$$\frac{H^{\prime }}{W}=\frac{W^{\prime }}{H}$$

This ensures that the aspect ratio remains constant, preventing image distortion.

## Limitations

The “Tomato Leaf Dataset” may not large enough to capture the full variability of tomato leaf diseases, potentially impacting the generalizability of the model. Since the images were collected from a limited number of tomato fields or regions, the dataset does not represent the diversity of tomato leaf diseases across different climates and geographical locations. Some of the limitations are as follows:

• The dataset may not accurately represent the diversity of tomato leaf diseases due to geographical limitations, which include factors like soil type, height, sea level, and climate, which can impact tomato leaves in various environmental situations.
• Despite efforts to minimize human subjectivity in gathering tomato leaf photos, potential biases and mistakes may have impacted the accuracy of our dataset, highlighting the need for further research and improvement.
• Multiple disease-infected leaves pose a challenge for the model's categorization, potentially limiting its accuracy and impacting its performance and reliability in practical applications.

## Ethics Statement

During data collection, neither plants nor animals contracted any infections. The dataset collected with the consent of tomato farmers, adhered to ethical guidelines. The aim was to advance knowledge in agricultural disease detection and management, not for commercial exploitation, and the data is intended for research purposes only.

## Credit Author Statement

**Ahmed Imtiaz:** Conceptualization, Methodology, Data Curation, Writing - original draft. **Fahad Bin Islam Swapnil:** Writing – Original draft, Validation. **Syed Rayhan Masud:** Writing - Original draft, Background & Editing. **Debajyoti Karmaker:** Supervision, Writing - Review & Editing.

## Data Availability

Mendeley DataTomato Leaf Dataset : A dataset for multiclass disease detection and classification (Original data).
